# Sample Preconcentration Utilizing Nanofractures Generated by Junction Gap Breakdown Assisted by Self-Assembled Monolayer of Gold Nanoparticles

**DOI:** 10.1371/journal.pone.0126641

**Published:** 2015-05-13

**Authors:** Chun-Ping Jen, Tamara G. Amstislavskaya, Kuan-Fu Chen, Yu-Hung Chen

**Affiliations:** 1 Department of Mechanical Engineering and Advanced Institute of Manufacturing with High-Tech Innovations, National Chung Cheng University, Chia Yi, Taiwan, R.O.C; 2 Laboratory of Experimental Models of Emotional Pathology, Scientific Research Institute of Physiology and Basic Medicine, Novosibirsk, Russia; 3 Department of Medicine, National Cheng-Kung University, Tainan, Taiwan, R.O.C; 4 Department of Biochemistry and Molecular Biology, National Cheng-Kung University, Tainan, Taiwan, R.O.C; Northeastern University, UNITED STATES

## Abstract

The preconcentration of proteins with low concentrations can be used to increase the sensitivity and accuracy of detection. A nonlinear electrokinetic flow is induced in a nanofluidic channel due to the overlap of electrical double layers, resulting in the fast accumulation of proteins, referred to as the exclusion-enrichment effect. The proposed chip for protein preconcentration was fabricated using simple standard soft lithography with a polydimethylsiloxane replica. This study extends our previous paper, in which gold nanoparticles were manually deposited onto the surface of a protein preconcentrator. In the present work, nanofractures were formed by utilizing the self-assembly of gold-nanoparticle-assisted electric breakdown. This reliable method for nanofracture formation, involving self-assembled monolayers of nanoparticles at the junction gap between microchannels, also decreases the required electric breakdown voltage. The experimental results reveal that a high concentration factor of 1.5×10^4^ for a protein sample with an extremely low concentration of 1 nM was achieved in 30 min by using the proposed chip, which is faster than our previously proposed chip at the same conditions. Moreover, an immunoassay of bovine serum albumin (BSA) and anti-BSA was carried out to demonstrate the applicability of the proposed chip.

## Introduction

In biochemical analysis, numerous strategies, such as field-amplified sample stacking (FASS) [1,2], isotachophoresis (ITP) [3], isoelectric focusing (IEF) [4], temperature gradient focusing (TGF) [5], nanofilters [6], and nanoporous membrane/nanochannel techniques [7–10], have been broadly exploited to preconcentrate the low-concentration protein samples [11] and described in detail previously [12]. FASS, ITP, and IEF procedures are complex, and at least two types of buffer solution are needed. The operation of TGF is based on the electrophoretic velocity of an analyte changing as a function of temperature and thus requires precise temperature control. An approach utilized the filtering effect to stack analytes on one side of a nanoporous membrane or nanochannel with pore sizes or channel dimensions smaller than that of the analytes has been investigated [6]. In addition, analytes may be concentrated in nanopores or nanochannels by the exclusion-enrichment effect [13], which is caused by the overlap of electrical double layers in a nanofluidic channel, an inherent ion-permselectivity allows counter ions to pass through the nanochannel, but repels coions from the nanochannel. Then, the polarization of concentrations produces the ionic depletion effect on the anodic side of the nanochannel under an electric field. A nonlinear electrokinetic flow generated by the unbalanced distribution of ions results in the fast accumulation of proteins in front of the induced ionic depletion zone, referred to as the exclusion-enrichment effect [13]. The fabrication of nanofluidic channels is the key technique to create the exclusion-enrichment effect and simple buffer systems employed is the major advantage of manipulation [14]. A number of available approaches for the building nanochannels/nanopores have been reported [15]. Standard photolithography and high-accuracy etching techniques [16] have been used to fabricate a microdevice with nanofluidic channels. An extended space charge region was generated for electrokinetically collecting and trapping proteins, with concentration factors of as high as 10^6^–10^8^ [7]. However, this method is time-consuming and costly [11]. Porous membranes have received attention because commercially available membranes can be integrated easily onto microchips. An accumulation factor of 10^5^–10^6^ has been achieved in a polydimethylsiloxane (PDMS) microdevice integrated with polycarbonate track-etched (PCTE) membranes with 10-nm nanopores [6]. In a microfluidic sample preconcentration system with a highly ion-conductive, charge-selective polymer, poly-AMPS (2-acrylamido-2-methyl-1-propanesulfonicacid), a concentration factor of 10^3^ was reached in 20 min [8]. Another highly porous ion-selective material, Nafion resin, has been integrated with PDMS/glass-based microfluidic chips. A surface pattern printed on a submicron-thick Nafion film in PDMS/glass-based microfluidic chips was proposed for multiplexed proteomic sample preconcentration, achieving a concentration factor of 10^4^ in 5 min [8]. The PDMS gap created by mechanical cutting was filled using a Nafion polymer solution and self-sealed due to its flexibility [10]. The preconcentration of β-phycoerythrin proteins in large channels was achieved with a concentration factor of up to 10^4^. A massive array of 128 parallel nanofluidic concentration microdevices with Nafion nanoporous junctions for high-throughput biomolecule detection was proposed to substantially increase the dynamic range of immunoassays [17]. Although using photopolymerization might overcome the problem of liquid leakage, a complex optical setup and careful operation are required to complete the process [18]. Thus, a simpler technique for fabricating nanochannels or nanofractures that adopts the junction gap electric breakdown between two PDMS microchannels [19] was reported. Nanogaps form between the PDMS microchannels when a high voltage is applied. A direct-current (DC) voltage of 1000 V was applied between 40-μm-wide microchannels; the corresponding electric field of 25 V/μm was slightly greater than the dielectric strength of PDMS (21 V/μm), thus creating a nanogap with a depth of approximately 80 nm. Lee et al. demonstrated a concentration factor of as high as 10^4^ within 1 h [19]. Spontaneously formed nanochannels underneath the PDMS layer were reversibly and weakly bonded to a glass substrate [20]. A concentration factor of 10^3^ to 10^6^ was achieved in 30 min on a microchip with chevron-shaped microchannels in a mirror-image orientation. However, the use of reversible bonding between PDMS and a glass substrate is less robust than the permanent bonding obtained using oxygen-plasma treatment. A microchip with two printed V-shaped microchannels in a mirror-image orientation separated by a 100-μm gap has been reported [18]. Nanofractures were formed by inducing an electric breakdown using a high electric field, with concentration factors of 10^3^–10^5^ achieved. Comparing to the previously described porous membrane-based technique, the nanogaps were fabricated without using special reagents or materials; instead, a junction gap electric breakdown was induced. However, the required voltage for initiating an electric breakdown is high, and the cross-sectional areas of the nanogaps are smaller than those of a porous membrane [11]. Our previous study [12] reported a method for forming nanofractures by deposing gold nanoparticles at the junction gap between microchannels to reduce the required electrical breakdown voltage. A 100-nL droplet of liquid containing 1 nM gold nanoparticles was manually dropped onto the junction gaps; the necessary voltage was 36% of that required for chips without nanoparticle deposition. A sample of proteins with an extremely low concentration of 1 nM was concentrated to 1.5×10^4^-fold in 60 min. The formation of nanofractures via the self-assembly of gold nanoparticles to facilitate electric breakdown is investigated in the present study. The exclusion-enrichment effect in a nanofluidic channel is adopted to preconcentrate proteins. The proposed nanofluidic chip for the preconcentration of proteins was fabricated using simple standard soft lithography with a PDMS replica. The method for nanofracture formation, which involves the use of self-assembled monolayers (SAMs) of nanoparticles at the junction gap between microchannels, can be well controlled and is reliable. DC current measurements as a function of applied voltage (*I-V* curves) were made to determine the electrical properties between the junction gaps in the presence of the self-assembly of gold nanoparticles. An immunoassay of bovine serum albumin (BSA) and anti-BSA was performed to demonstrate the applicability of the proposed preconcentrator.

## Experimental Section

### Design and fabrication of microchips

**Fig 1:**
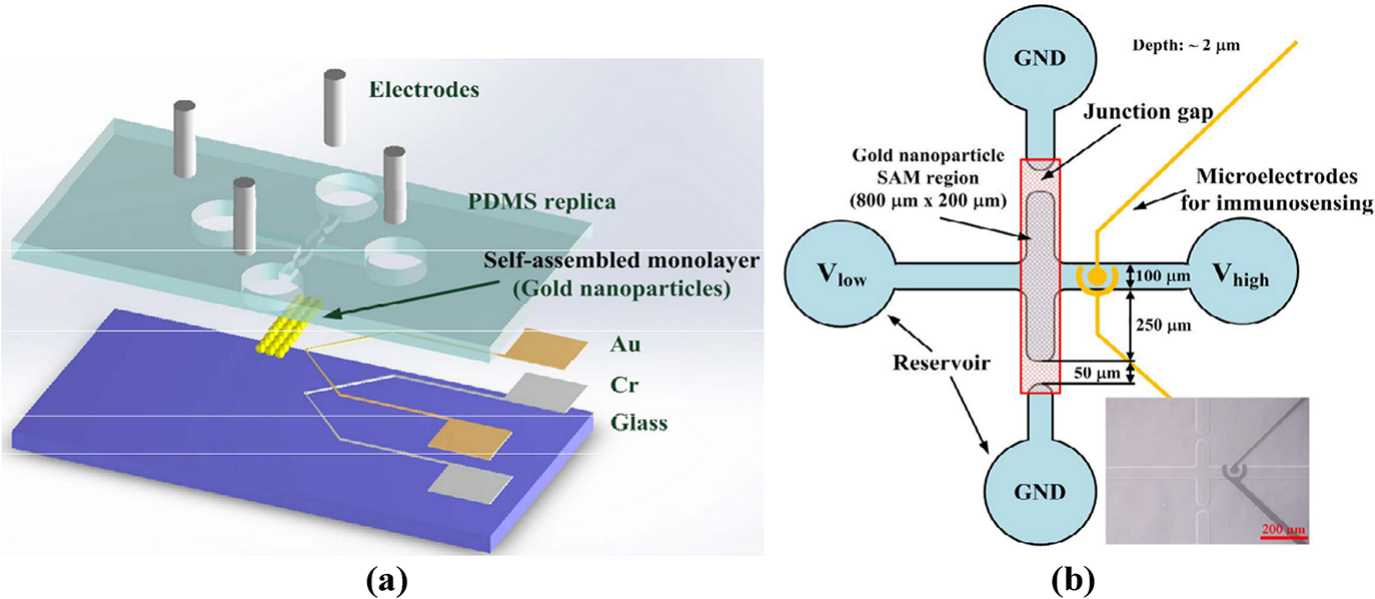
(a) Schematic illustration and (b) layout of protein preconcentrating microchip with self-assembly of gold nanoparticles (optical microscopy image shown in inset).

**Fig 2:**
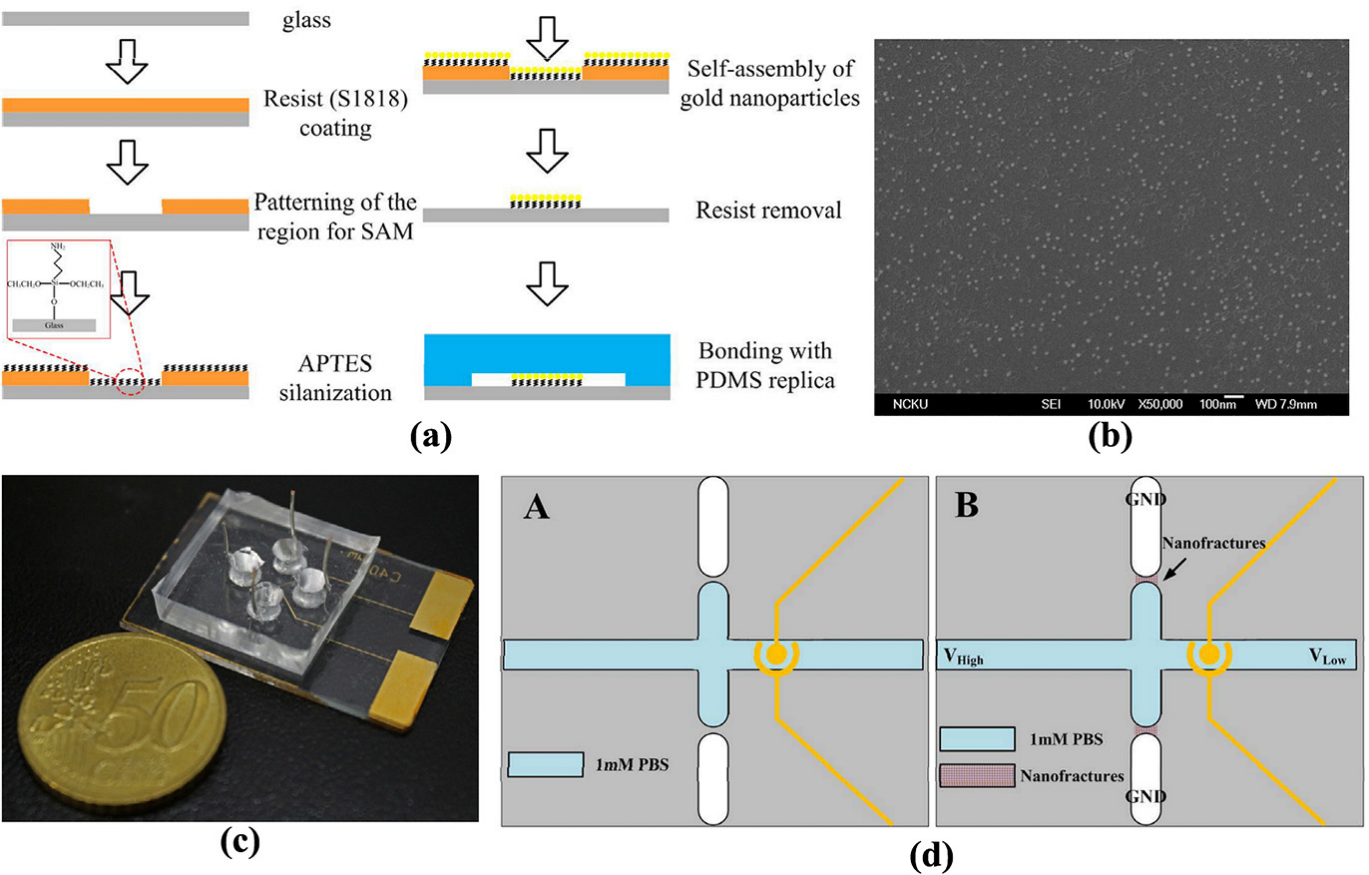
(a) Schematic diagram of patterned APTES silanization and gold nanoparticle self-assembly processes; (b) SEM image of SAM of 0.5 nM gold nanoparticles; (c) image of fabricated microchip; (d) illustrations of nanofracture formation for protein preconcentration.

A schematic diagram and layout of the proposed protein preconcentrator are shown in Fig 1. PDMS, which is biocompatible and transparent, was adopted for fabricating the micro/nanofluidic channels in the chip for protein preconcentration. Two junction gaps were designed along the main microchannel for the formation of nanofractures via electric breakdown. The region for the self-assembly of gold nanoparticles was patterned on the glass substrate, as shown in Fig 1A. A pair of microelectrodes was placed in the concentration region to demonstrate the immunoassay of BSA and anti-BSA, as shown in Fig 1A. The dimensions of the micro/nanofluidic channels are depicted in Fig 1B and the optical microscopy image is shown in the inset. The depth and width of the main microchannel are 2 and 100 μm, respectively. The width of the junction gap is 50 μm. To reduce the alignment difficulty during the bonding process of the PDMS replica with the glass substrate, the region of gold self-assembly was designed to be 200 μm × 800 μm under the junction gaps and the vertical main microchannel to ensure that it covered the junction gaps. The mold master for defining the microchannels was fabricated by spinning S1818 (Rohm and Haas Electronic Materials LLC, Philadelphia, PA, USA) on a silicon wafer (approximately 2 μm thick). A 5:1 weight mixture of PDMS prepolymer and curing agent (Sylgard-184 Silicone Elastomer Kit, Dow Corning, Midland, MI, USA) was poured and cured on the mold master to replicate the microchannel. After the PDMS replica had been peeled away, the inlet and outlet ports were created using a puncher. The region for the self-assembly of gold nanoparticles on the glass substrate was patterned using standard photolithography with S1818 photoresist. Silanization on the glass was achieved using 6 μL of 0.1% v/v (3-aminopropyl)triethoxysilane (APTES)/H_2_O solution for 1 min, as shown in Fig 2A. The glass substrate was then nitrogen-dried after being rinsed in deionized (DI) water, and reacted for 1 h in 30 μL of liquid containing various concentrations of gold nanoparticles (0.5, 1.0, and 2.0 nM) for self-assembly. Subsequently, the glass underwent multiple rinses in acetone, methanol, and DI water to eliminate the photoresist. The gold nanoparticles used in this study were prepared based on the procedure reported by Natan [21](18). Briefly, a solution of tetrachloroauric acid was synthesized by dissolving 39.37 mg of HAuCl_4_ 3H_2_O in 100 mL of distilled DI water. After the solution was heated to boiling, 10 mL of a 38.8 mM aqueous sodium citrate solution was added to it under vigorous stirring to form a claret solution. The mean diameter of gold nanoparticles was 13.7±0.8 nm. The original particle concentration was approximately10 nM. The solutions with gold colloids were diluted to 0.5, 1.0, and 2.0 nM, respectively, for the self-assembly of nanoparticles on the glass substrate after silanization. Fig 2B shows a scanning electron microscopy (SEM) image of the SAM of 0.5 nM gold nanoparticles. After the nanoparticles had self-assembled in the designed region, the PDMS replica was bonded to the glass substrate using oxygen-plasma treatment in anO_2_ plasma cleaner (PDC-32G, Harrick Plasma Corp., Ithaca, NY, USA).Four electrodes were then inserted into the reservoirs to apply the required voltage in the experiments. The fabricated chip is shown in Fig 2C.

### Formation of nanofractures and protein preconcentration

Nanofractures were created between microchannels via junction gap breakdown. Phosphate-buffered saline (PBS; 1 mM, pH 7.4) solution was used for the buffer system in this study. A DC voltage was applied to the two anodic side reservoirs while the other reservoirs were grounded, as shown in Fig 2D, initiating electric breakdown and thus forming nanofractures. The *I-V* curves before and after the creation of nanofractures were measured using a source measure unit (B2902A Precision Source/Measurement Unit, Agilent Technologies, Santa Clara, CA, USA) to determine the electrical characteristics of nanofractures. Fluorescein isothiocyanate (FITC)-labeled BSA (Sigma-Aldrich, St. Louis, MO, USA), diluted in 1 mM PBS at concentrations of 1 nM and 100 nM, was allowed to fill the microchannel via capillary force to demonstrate the on-chip protein preconcentration.

### Apparatus

A high-voltage power supply (Series 225, Bertan High Voltage Corp., Hicksville, NY, USA) was employed to provide the required voltages for both junction gap breakdown and protein preconcentration. The *I-V* curves for the region between the junction gaps before and after the application of a DC voltage for electric breakdown were measured using an Agilent B2902A Precision Source/Measurement Unit. Protein preconcentration was observed and recorded using an inverted fluorescence microscope (CKX41, Olympus, Tokyo, Japan) with a mounted CCD camera (DP71, Olympus, Tokyo, Japan) and connected to a computer running Olympus DP Controller image software. The fluorescence intensities emitted by enriched FITC-labeled BSA were quantified using ImageJ software (National Institutes of Health, Bethesda, Maryland, USA), which can assess the density of each pixel.

## Results and Discussion

**Fig 3:**
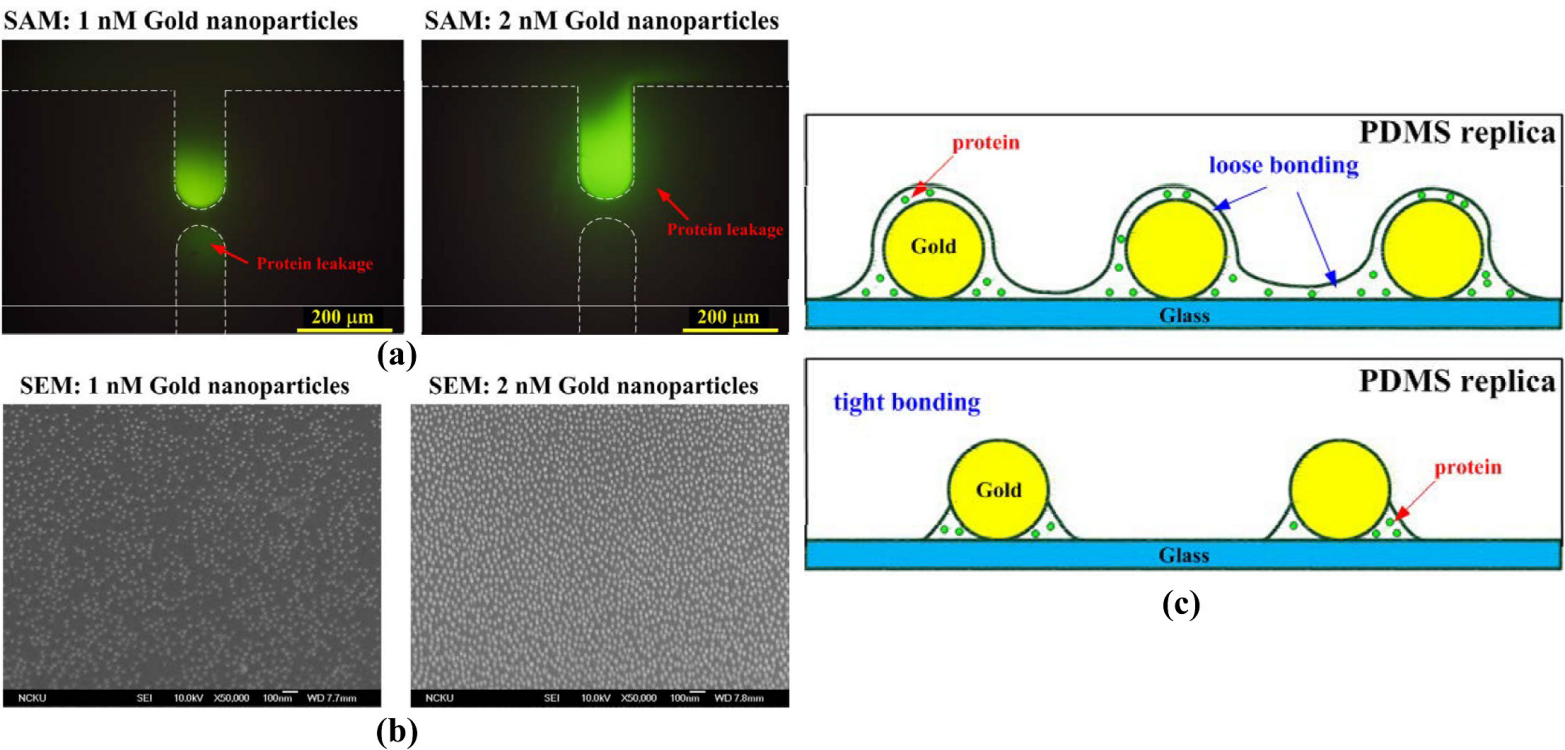
(a) Protein leakage due to insufficient bonding strength between PDMS replica and glass with SAM of 1.0 and 2.0 nM gold nanoparticles for self-assembly; (b) SEM images of SAM of 1.0 and 2.0 nM gold nanoparticles; (c) schematic illustration of loose and tight bonding between PDMS replica and glass with SAM of nanoparticles.

The solutions with 0.5, 1.0, and 2.0 nM gold nanoparticles were adopted for the self-assembly of nanoparticles on the glass substrate after silanization. When 1.0 and 2.0 nM gold nanoparticles were used, the strength of bonding between the PDMS replica and the glass with a SAM of nanoparticles was insufficiently high; therefore, the sample of proteins leaked out, as shown in Fig 3A. SEM images of the SAM of 1.0 and 2.0 nM gold nanoparticles are shown in Fig 3B. Loose bonding around the self-assembled nanoparticles occurred due to the higher density of nanoparticles underneath the junction gaps, which acted as a steric hindrance during PDMS bonding. However, a tight bonding formed with 0.5 nM gold nanoparticles because the lower nanoparticle density created more space for the complete bonding between PDMS and the glass substrate (Fig 3C).

**Fig 4:**
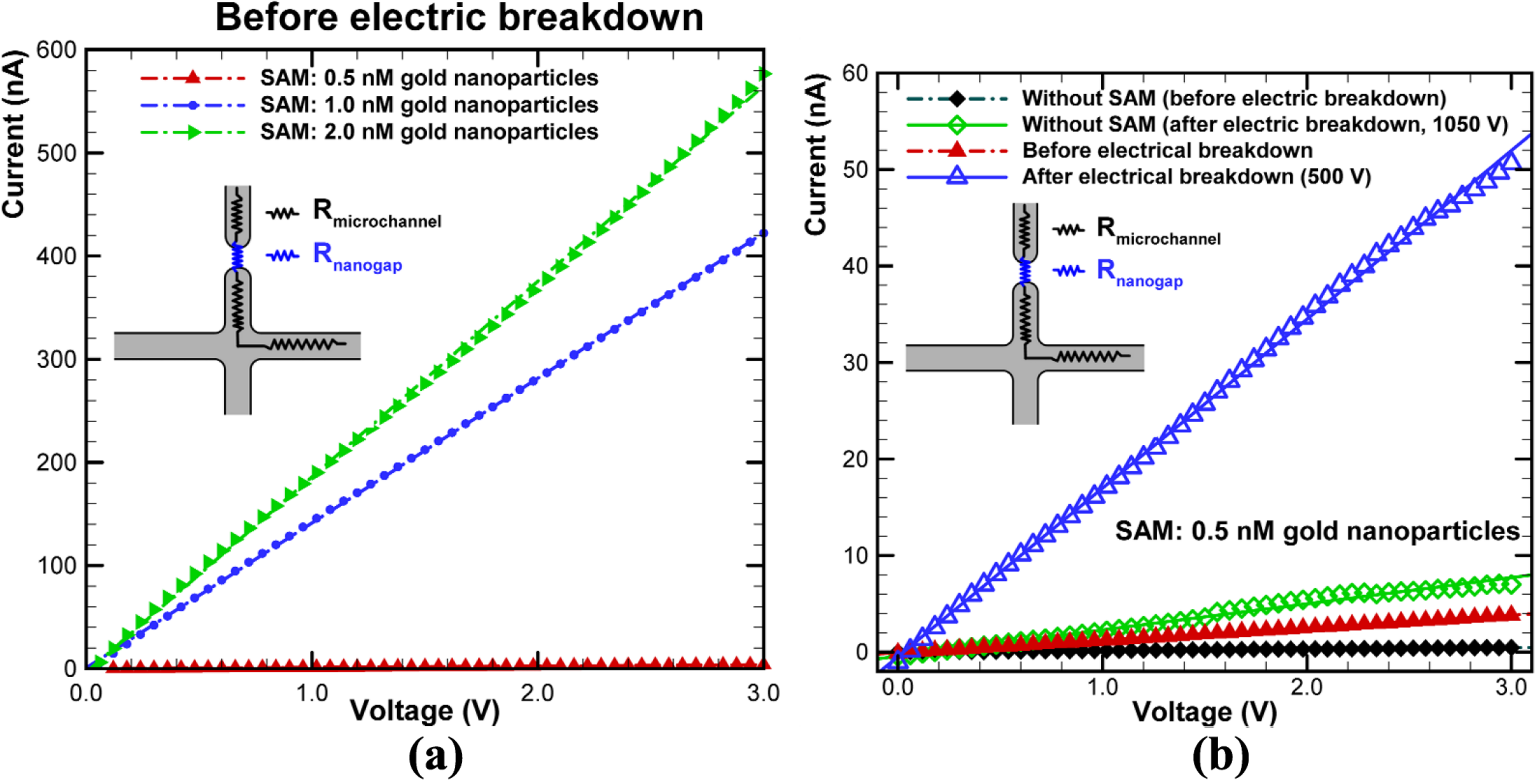
(a) Measured *I-V* curves for region between junction gaps before application of electric breakdown for cases with 0.5, 1.0, and 2.0 nM gold nanoparticles for self-assembly; (b) *I-V* curves before and after nanofracture formation for case with 0.5 nM gold nanoparticles for self-assembly compared with case without SAM. Simple equivalent circuit for measurement is shown in inset.

The conductivity of a rectangular-shaped nanochannel was measured by Stein et al. [22], who determined that the electrical conductance of channels becomes saturated at a value that is independent of both the salt concentration and the channel height under low-salt conditions. DC current measurements as a function of applied voltage (*I-V* curves) were performed in a previous study [19] to investigate the formation and dimensions of the nanogap using a relatively high concentration of KCl electrolyte (10^–1^ M). In the present study, DC current measurements between the junction gaps were performed in a high-salt (1.0 M KCl) electrolyte buffer system to determine the characteristics of nanofractures. The measured *IV* curves are shown in Fig 4. Gold nanoparticles self-assembled onto the surface of the protein preconcentrator. The existence of gold nanoparticles was expected to enlarge the electric current in the nanofluidic channel. *I-V* curves for the region between the junction gaps for 0.5, 1.0, and 2.0 nM gold nanoparticles used for self-assembly before the electric breakdown are shown in Fig 4A. When particle concentrations of 1.0 and 2.0 nM were employed, the currents substantially increased because of the loose bonding, in contrast to that for the 0.5 nM concentration. The *I-V* curves before and after nanofracture formation, when 0.5 nM gold nanoparticles was adopted for self-assembly, are shown in Fig 4B and compared to the case without a SAM. In the *I-V* curve for the case without a SAM before the electric breakdown, the current between the junction gaps was almost zero while the applied voltage from 0 up to 3.0 V, indicating that the resistance was infinite due to the absence of nanofractures. However, the electric current between the junction gaps increased when 0.5 nM gold nanoparticles self-assembled on the glass substrate, even though the electric breakdown had not yet been applied (as indicated by the red line with triangular symbols in Fig 4B). The electric current went through the conductive gold nanoparticles. The creation of nanogaps around self-assembled nanoparticles was inevitable because of the nanoparticles underneath the junction gaps, despite the tight bonding. These nanogaps create alternative current paths that increase the electric current, as shown in the *I-V* curve with triangular symbols in Fig 4B. When a DC voltage of 1050 V (the dielectric strength of a PDMS gap with a width of 50 μm) was applied to the chip without a SAM to initiate electric breakdown, the slope in measured *I-V* curve increased significantly, indicating that the electric current enhanced due to the formation of nanofractures. Various DC voltages for initiating the electric breakdown on the chip with a SAM were applied. A voltage of 500 V was adopted to create nanofractures for generating a depletion force sufficient for concentrating proteins based on a method of the quantitative evaluation of the depletion force [23]. The measured *I-V* curve indicated that the current was substantially larger than that for the chip without a SAM after electric breakdown. In our experiments, the solution with 0.5 nM nanoparticles for self-assembly yielded reliable results for bonding and protein preconcentration; hence, this particle concentration was applied in subsequent experiments. Nanofractures were created using applied voltages of as low as 500 V for 60 s when nanoparticles with a concentration of 0.5 nM self-assembled onto the glass substrate underneath the junction gaps.

**Fig 5:**
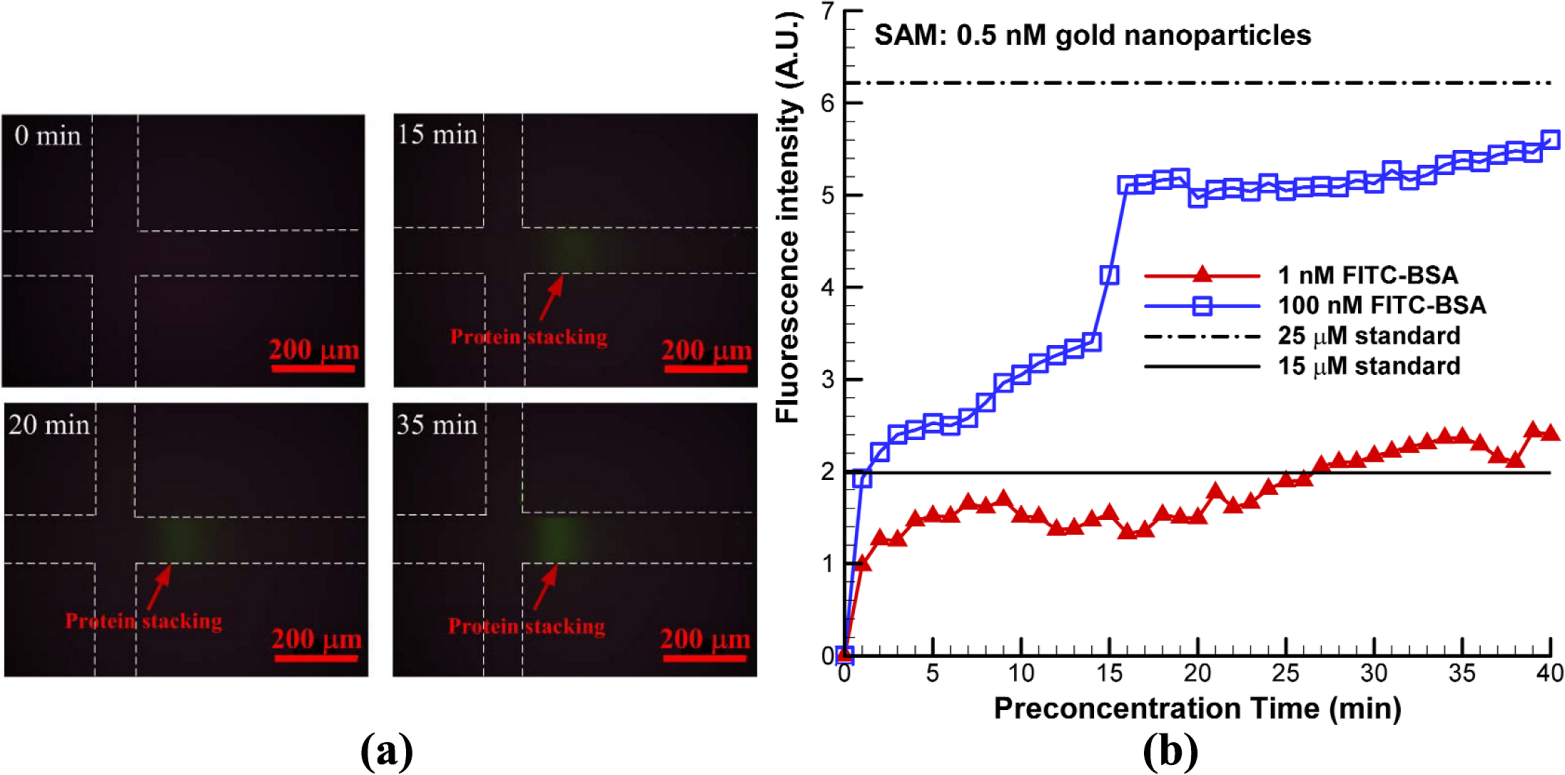
(a) Fluorescence images of 1 μM FITC-labeled BSA in 1 mM PBS solution (pH 7.4) taken at various time points; (b) concentration performance for initial protein concentrations of 1 nM and 100 nM. Chip with SAM of 0.5 nM gold nanoparticles at junction gaps had DC voltage of 500 V applied to it for 60 s to create nanofractures.

**Fig 6:**
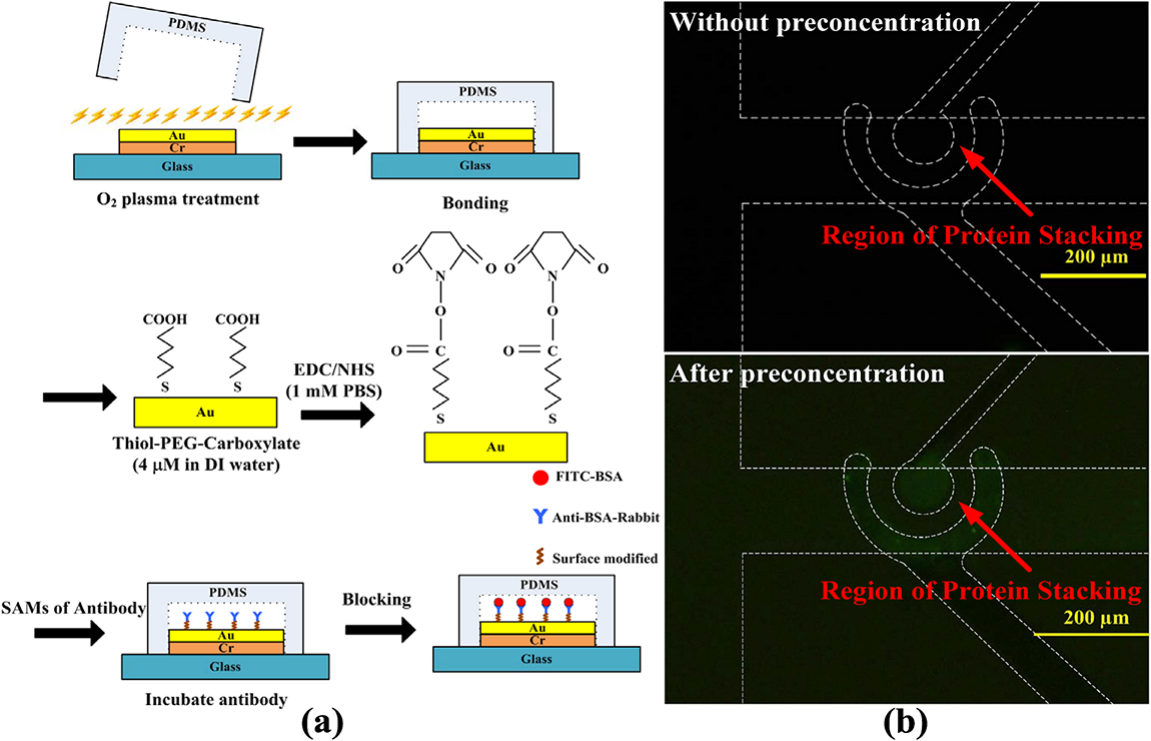
(a) Processes for modification of functional groups on microelectrode surface; (b) experimental results of immunoassay with and without protein preconcentration. Initial concentration of FITC-BSA was 10 μM. Reaction time for concentrating was 40 min (exposure time was 6 s).

After the nanofractures were generated, FITC-labeled BSA was diluted in a 1 mM PBS solution at concentrations of 1 nM, 100 nM, and 1 μM. It was then allowed to fill the microchannel via capillary force to perform the on-chip preconcentration of proteins. The operations of the electrokinetic protein preconcentration were presented in our previous work [12]. The depletion regions in the main vertical channel elongated when a DC voltage of 50 V was applied to the two anodic side reservoirs while the other reservoirs were grounded. The elongating depletion regions from the top and bottom junction gaps merged and extended to the main horizontal channel. A bias voltage of 46 V was set on the left anodic side to induce electroosmotic flow to accumulate proteins. Fluorescence images of 1 μM FITC-labeled BSA in 1 mM PBS solution taken at various time points are shown in Fig 5A. The results reveal that both the concentration of BSA and the size of the preconcentration area increased with time, which demonstrates that proteins can be concentrated using the proposed chip with SAMs of gold nanoparticles on a glass substrate underneath the junction gaps. The concentration of collected BSA proteins obtained from fluorescence intensity was quantified and averaged over a rectangular window using ImageJ software. The concentration performance for initial protein concentrations of 1 nM and 100 nM is plotted in Fig 5B. To estimate the final concentration, the fluorescence intensity of the standard sample solutions (15 and 25 μM) was measured. The results are shown in this figure. The experimental results indicate that the protein sample with a concentration of 100 nM was concentrated to approximately 25 μM in 40 min (approximately 250-fold the initial concentration). The sample with a concentration of 1 nM exceeded 15 μM in 30 min, indicating that a protein sample with an extremely low concentration of 1 nM can be concentrated to more than 1.5×10^4^-fold in 30 min. Moreover, an immunoassay of BSA and anti-BSA was performed to demonstrate the applicability of the proposed preconcentrator. The modification of the functional groups on the microelectrode surface is described below and shown in Fig 6A. After the PDMS microchannel was aligned to the microelectrode and bonded to O_2_ plasma-treated glass, 4 μM O-(2-Carboxyethyl)-O’-(2-mercaptoethyl)heptaethylene glycol (Sigma–Aldrich, St Louis, MO, USA) as a surface modification compound was injected into the microchannel and incubated for 12 h. The thiol groups form Au-S bonds on the surface of Au microelectrodes and produce a SAM after exposure to carboxyl groups. PBS (1 mM) was used to wash the microchannel after 12 h. Then, a mixture of 10 mg/mL EDC *N*-(3-Dimethylaminopropyl)-*N*-ethylcarbodiimide hydrochloride (EDC, Sigma–Aldrich, St Louis, MO, USA) and 5 mg/mL N-hydroxysuccinimide (NHS, Sigma–Aldrich, St Louis, MO, USA) (dissolved in 1 mM PBS) was injected and incubated for 1 h. This mixture acts as a coupling agent to activate the carboxyl group of thiolated polyethylene glycol into a reactive ester and form an amide bond with the amino group of anti-BSA on the SAM. The microchannel was washed after activation of the functional groups. Anti-BSA (0.2 mg/mL) was injected into the microchannel and incubated for 30 min to allow the formation of an amide bond with the SAM. After anti-BSA was conjugated to the SAM, the microchannel was washed and blocked with 1% biotin (1 mg/mL) for 30 min to minimize non-specific binding. Subsequently, the microchannel was washed with 1 mM PBS. The chip was then ready for concentration and immunoassay experiments. Experimental results of the immunoassay with and without protein preconcentration are shown in Fig 6B. The initial concentration of FITC-BSA was 10 μM. The reaction time was 40 min and the exposure time was 6 s. This experimental result demonstrated that the immunoassay on the microelectrodes could be performed successfully in the proposed microchip. The performance of the case with preconcentration was much better than that without preconcentration. This experiment also shows the capability of the proposed microchip for immunoassays after protein preconcentration. The interaction between antigen and antibody for immunoassay is designed on the recognition of protein tertiary structure. As shown on the analysis results, the tertiary structure of BSA proteins does not significantly change after the procedure of preconcentration. The protein structure still be kept and qualified to recognize its antibody. It also demonstrates that the proposed preconcentration microchip is capable of immunoassay applications.

## Conclusion

This paper proposed a method that involves using SAMs of nanoparticles at the junction gaps between microchannels for forming nanofractures, which reduce the required electric breakdown voltage. In our experiments, the solution with 0.5 nM nanoparticles for self-assembly after silanization yielded reliable results for bonding and protein preconcentration. Nanofractures were created using applied voltages of as low as 500 V for 60 s when nanoparticles self-assembled on the glass substrate underneath the junction gaps. The experimental results reveal that a protein sample with a concentration of 100 nM became concentrated to approximately 250-fold the initial concentration. Moreover, a protein sample with an extremely low initial concentration of 1 nM was concentrated to more than1.5×10^4^-fold in 30 min. The concentration factor obtained with the proposed design is comparable to those of existing devices; however, the required voltage is substantially lower. The electrokinetic preconcentration of proteins was demonstrated using nanofractures generated by nanoparticle-assisted electric breakdown at the junction gaps. Furthermore, an immunoassay of BSA and anti-BSA was performed, with results demonstrating the applicability of the proposed microchip.

## References

[1] Chun H, Chung TD, Ramsey JM. High yield sample preconcentration using a highly ion-conductive charge-selective polymer. Anal Chem. 2010;82: 6287–6292. 10.1021/ac101297t 20575520 PMC3125590

[2] Lichtenberg J, Verpoorte E, de Rooij NF. Sample preconcentration by field amplication stacking for microchip-based capillary electrophoresis. Electrophoresis. 2001. pp. 258–271. 10.1002/1522-2683(200101)22:2<258::AID-ELPS258>3.0.CO;2-4 11288893

[3] Jung B, Bharadwaj R, Santiago JG. On-chip millionfold sample stacking using transient isotachophoresis. Anal Chem. 2006;78: 2319–2327. 10.1021/ac051659w 16579615

[4] Hofmann O, Che D, Cruickshank KA, Muller UR. Adaptation of capillary isoelectric focusing to microchannels on a glass chip. Anal Chem. 1999;71: 678–686. 10.1021/ac9806660 9989385

[5] Ross D, Locascio LE. Microfluidic temperature gradient focusing. Anal Chem. 2002;74: 2556–2564. 10.1021/ac025528w 12069237

[6] Wu D, Steckl AJ. High speed nanofluidic protein accumulator. Lab Chip. 2009;9: 1890–1896. 10.1039/b823409d 19532964

[7] Wang YC, Stevens AL, Han J. Million-fold preconcentration of proteins and peptides by nanofluidic filter. Anal Chem. 2005;77: 4293–4299. 10.1021/ac050321z 16013838

[8] Lee JH, Song Y-A, Han J. Multiplexed proteomic sample preconcentration device using surface-patterned ion-selective membrane. Lab Chip. 2008;8: 596–601. 10.1039/b717900f 18369515 PMC2394188

[9] Liu V, Song Y-A, Han J. Capillary-valve-based fabrication of ion-selective membrane junction for electrokinetic sample preconcentration in PDMS chip. Lab Chip. 2010;10: 1485–1490. 10.1039/b923214a 20480116 PMC2926974

[10] Sung JK, Han J. Self-sealed vertical polymeric nanoporous-junctions for high-throughput nanofluidic applications. Anal Chem. 2008;80: 3507–3511. 10.1021/ac800157q 18380489 PMC2750818

[11] Lin CC, Hsu JL, Lee GB. Sample preconcentration in microfluidic devices. Microfluidics and Nanofluidics. 2011. pp. 481–511. 10.1007/s10404-010-0661-9

[12] Jen C-P, Amstislavskaya TG, Kuo C-C, Chen Y-H. Protein preconcentration using nanofractures generated by nanoparticle-assisted electric breakdown at junction gaps. PLoS One. 2014;9: e102050 10.1371/journal.pone.0102050 25025205 PMC4098899

[13] Pu Q, Yun J, Temkin H, Liu S. Ion-enrichment and ion-depletion effect of nanochannel structures. Nano Lett. 2004;4: 1099–1103. 10.1021/nl0494811

[14] Song S, Singh AK, Kirby BJ. Electrophoretic concentration of proteins at laser-patterned nanoporous membranes in microchips. Anal Chem. 2004;76: 4589–4592. 10.1021/ac0497151 15283607

[15] Duan C, Wang W, Xie Q. Review article: Fabrication of nanofluidic devices. Biomicrofluidics. 2013;7 10.1063/1.4794973 PMC3612116 23573176

[16] Mao P, Han J. Fabrication and characterization of 20 nm planar nanofluidic channels by glass-glass and glass-silicon bonding. Lab Chip. 2005;5: 837–844. 10.1039/b502809d 16027934

[17] Ko SH, Kim SJ, Cheow LF, Li LD, Kang KH, Han J. Massively parallel concentration device for multiplexed immunoassays. Lab Chip. 2011;11: 1351–1358. 10.1039/c0lc00349b 21321747

[18] Yu H, Lu Y, Zhou Y, Wang F, He F, Xia X. A simple, disposable microfluidic device for rapid protein concentration and purification via direct-printing. Lab Chip. 2008;8: 1496–1501. 10.1039/b802778a 18818804

[19] Jeong HL, Chung S, Sung JK, Han J. Poly(dimethylsiloxane)-based protein preconcentration using a nanogap generated by junction gap breakdown. Anal Chem. 2007;79: 6868–6873. 10.1021/ac071162h 17628080 PMC2590669

[20] Kim SM, Burns MA, Hasselbrink EF. Electrokinetic protein preconcentration using a simple glass/poly(dimethylsiloxane) microfluidic chip. Anal Chem. 2006;78: 4779–4785. 10.1021/ac060031y 16841895

[21] Grabar KC, Freeman RG, Hommer MB, Natan MJ. Preparation and Characterization of Au Colloid Monolayers. Anal Chem. 1995;67: 735–743. 10.1021/ac00100a008

[22] Stein D, Kruithof M, Dekker C. Surface-Charge-Governed Ion Transport in Nanofluidic Channels. Phys Rev Lett. 2004;93: 035901 10.1103/PhysRevLett.93.035901 15323836

[23] Chiang P-J, Kuo C-C, Zamay TN, Zamay AS, Jen C-P. Quantitative evaluation of the depletion efficiency of nanofractures generated by nanoparticle-assisted junction gap breakdown for protein concentration. Microelectron Eng. 2014;115: 39–45. 10.1016/j.mee.2013.10.024

